# Familial Hypercholesterolemia Mimicking Rheumatoid Arthritis: A Rare Case From Pakistan

**DOI:** 10.7759/cureus.86207

**Published:** 2025-06-17

**Authors:** Sonia Golani, Sulhera Khan, Zara Saeed, Humaira Talat, Nazish Shah

**Affiliations:** 1 Dermatology, Dow University of Health Sciences, Civil Hospital Karachi, Karachi, PAK; 2 Internal Medicine, Jinnah Postgraduate Medical Centre, Karachi, PAK

**Keywords:** corneal arcus, familial hypercholestrolemia, rheumatoid arthritis, seronegative rheumatoid arthritis, tendon xanthoma

## Abstract

Familial hypercholesterolemia (FH) is a genetically inherited lipid disorder characterized by markedly elevated levels of low-density lipoprotein cholesterol (LDL-C), leading to premature cardiovascular disease and distinctive cutaneous manifestations such as tendon xanthomas and corneal arcus. We present the case of a young Pakistani woman with extensive yellowish plaques over her chest, hands, feet, and periocular area, alongside progressive deformities of the fingers and toes that clinically resembled rheumatoid arthritis, which developed over the course of four years. Laboratory investigations revealed significantly elevated total and LDL cholesterol, while autoimmune markers including rheumatoid factor (RF) and anti-CCP were negative. Radiographic imaging demonstrated soft tissue swelling, reduced joint spaces, and features of acro-osteolysis in the affected digits, suggestive of xanthomatous infiltration. The diagnosis of FH was established using clinical criteria, including the Simon-Broome criteria, Dutch Lipid Clinic Network (DLCN), and MEDPED scores, in the absence of genetic testing due to limited access. The patient was initiated on high-dose statin therapy with dietary and lifestyle modifications, and parents were offered genetic counselling. Although Proprotein Convertase Subtilisin/Kexin Type 9 Inhibitors (PCSK9) therapy was considered, its availability and cost posed significant barriers. This case highlights the critical role of physical findings and diagnostic scoring tools in identifying FH in resource-limited settings and adds to the scarce literature by reporting a rare rheumatoid-like presentation of FH, the first of its kind from Pakistan.

## Introduction

Familial hypercholesterolemia (FH) is an inherited autosomal dominant disorder characterized by markedly elevated levels of total cholesterol (TC) and low-density lipoprotein cholesterol (LDL-C), predisposing individuals to premature cardiovascular disease [1]. According to the meta-analysis, the global prevalence of FH is estimated at 1 in 250 individuals for heterozygous FH (HeFH) and approximately 1 in 300,000 to 1 in 1,000,000 for the homozygous variant (HoFH) [2]. Despite its relatively high prevalence, its prevalence is unknown in 90% of countries of the world, reflecting a significant gap in diagnosis and reporting [3].

The pathogenesis primarily involves mutations in genes affecting LDL metabolism. The most implicated gene is the low-density lipoprotein receptor (LDLR), accounting for over 80% of cases. Other less common mutations include those in apolipoprotein B (ApoB), proprotein convertase subtilisin/kexin type 9 (PCSK9), and LDL receptor adaptor protein 1 (LDLRAP1), the latter being associated with an autosomal recessive form of FH [4].

Clinically, FH presents with tendon xanthomas, corneal arcus, and premature atherosclerosis, which can result in early-onset myocardial infarction, cerebrovascular disease, or sudden cardiac death [5]. However, atypical manifestations can occur, particularly in severe or untreated cases.

We report the case of a 26-year-old woman presenting with classical cutaneous findings of FH: tendinous xanthomas and corneal arcus along with rheumatoid arthritis-like deformities of the hands and feet, a rare manifestation. These deformities are presumed to result from the deposition of lipid-laden macrophages (foam cells) in the soft tissues and periarticular areas, leading to joint stiffness and functional limitations that can mimic chronic inflammatory arthropathies such as rheumatoid arthritis [3].

## Case presentation

A 26-year-old married woman, a housewife and resident of interior Sindh, presented to a tertiary care hospital in Karachi, Pakistan, with complaints of multiple skin-colored to yellow swellings involving the hands, feet, and periocular area. She also reported similar plaque-like lesions on the chest and arms, progressively developing over the past four years. The lesions initially appeared as small papules near the medial canthus of both eyes and gradually increased in size, coalescing into larger, raised papules and plaques around the eyes. These lesions were painless and non-pruritic. Over the same time period, similar lesions appeared on the antecubital regions of both forearms. She also noted skin-colored swellings on the thighs, hands, and feet, particularly involving the digits. These digital swellings were associated with pain, causing mild stiffness and restricted joint movement. She denied experiencing shortness of breath, chest pain, or dizziness. Her drug history was unremarkable. On further inquiry, family history revealed that her mother had similar asymptomatic cutaneous lesions and did not seek medical attention. Her maternal uncle reportedly had similar lesions and died suddenly at the age of 40, likely due to sudden cardiac death. There was no history of addiction, recent travel, or significant personal history.

**Figure 1:**
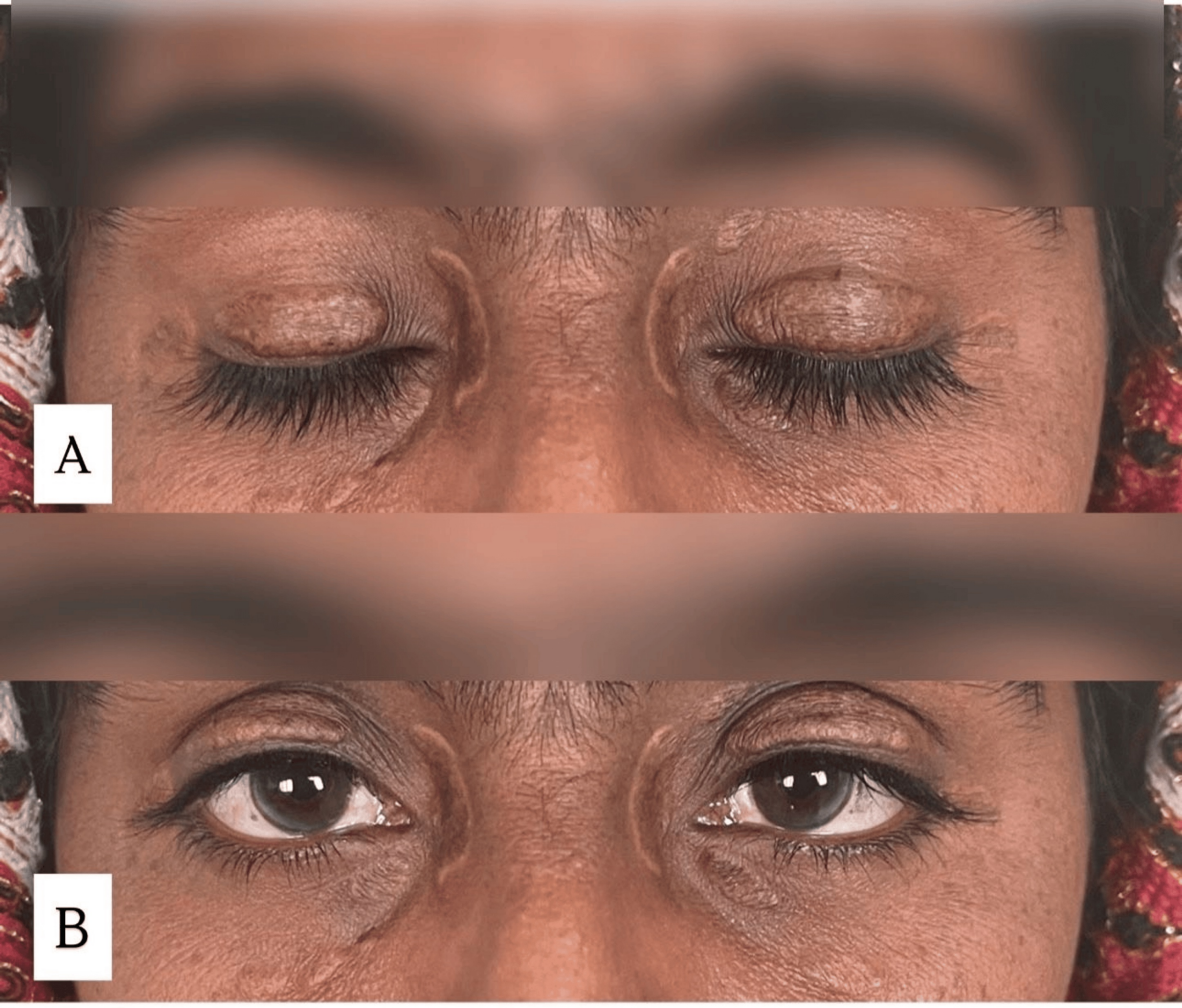
Skin-colored to yellow xanthelasma palpebrarum (XP) in upper eyelids (A) and medial canthus (B)

On general physical examination, the patient was alert and oriented, with stable vital signs and no signs of systemic distress. Body mass index (BMI) was 24.5 kg/m². No pallor, cyanosis, clubbing, or lymphadenopathy was noted. Dermatological examination revealed multiple yellowish papules and plaques around the periocular region, predominantly over the medial canthus and upper eyelids (Figure 1). These lesions were soft to touch, non-tender, and had gradually coalesced into larger plaques.

**Figure 2:**
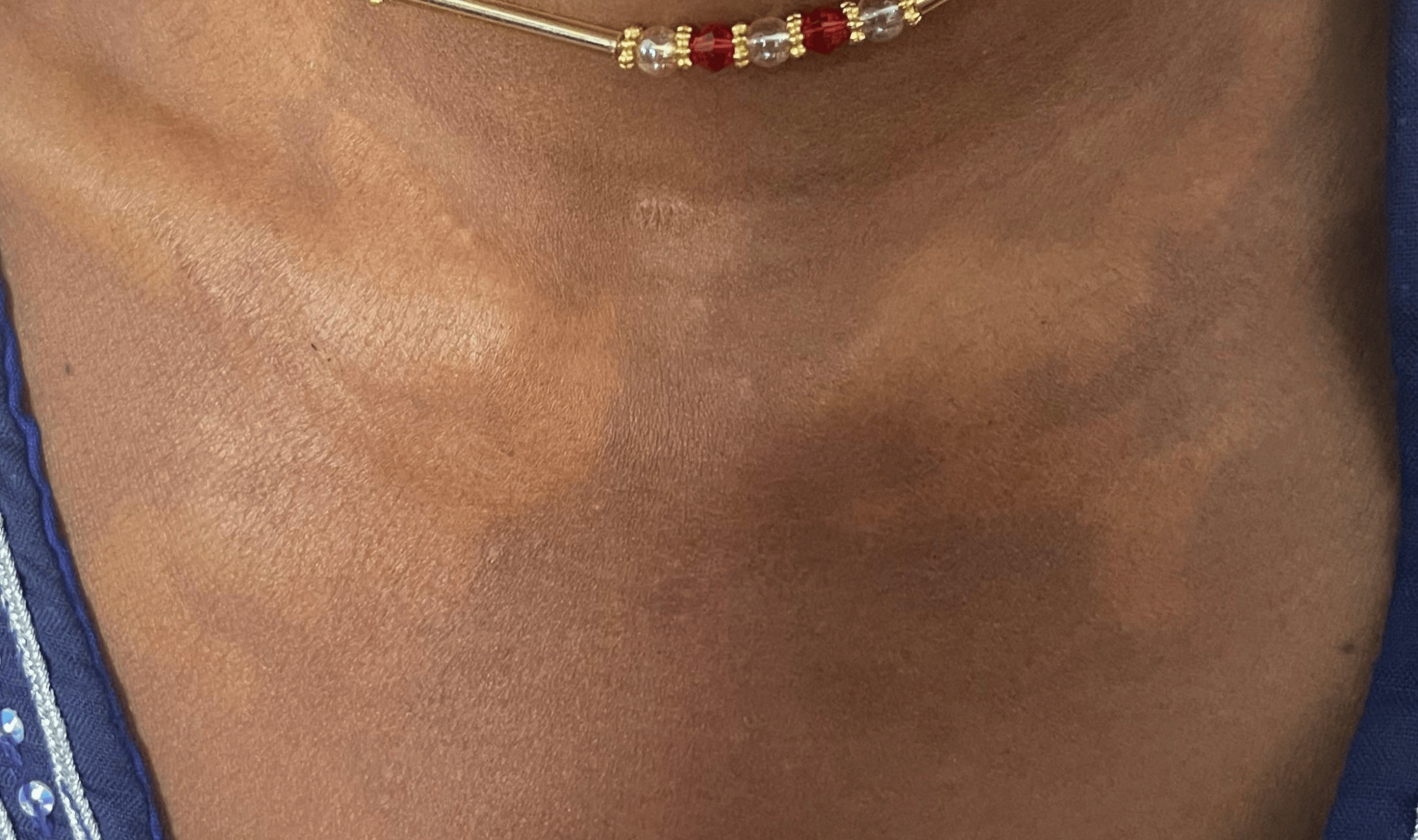
Large, irregular, yellow-colored plaques on the anterior chest at the level of the clavicles

On the antecubital fossae of both forearms, there were well-demarcated yellowish plaques interspersed with multiple open and closed comedones, giving a mixed papular-comedonal appearance (Figure 2).

**Figure 3:**
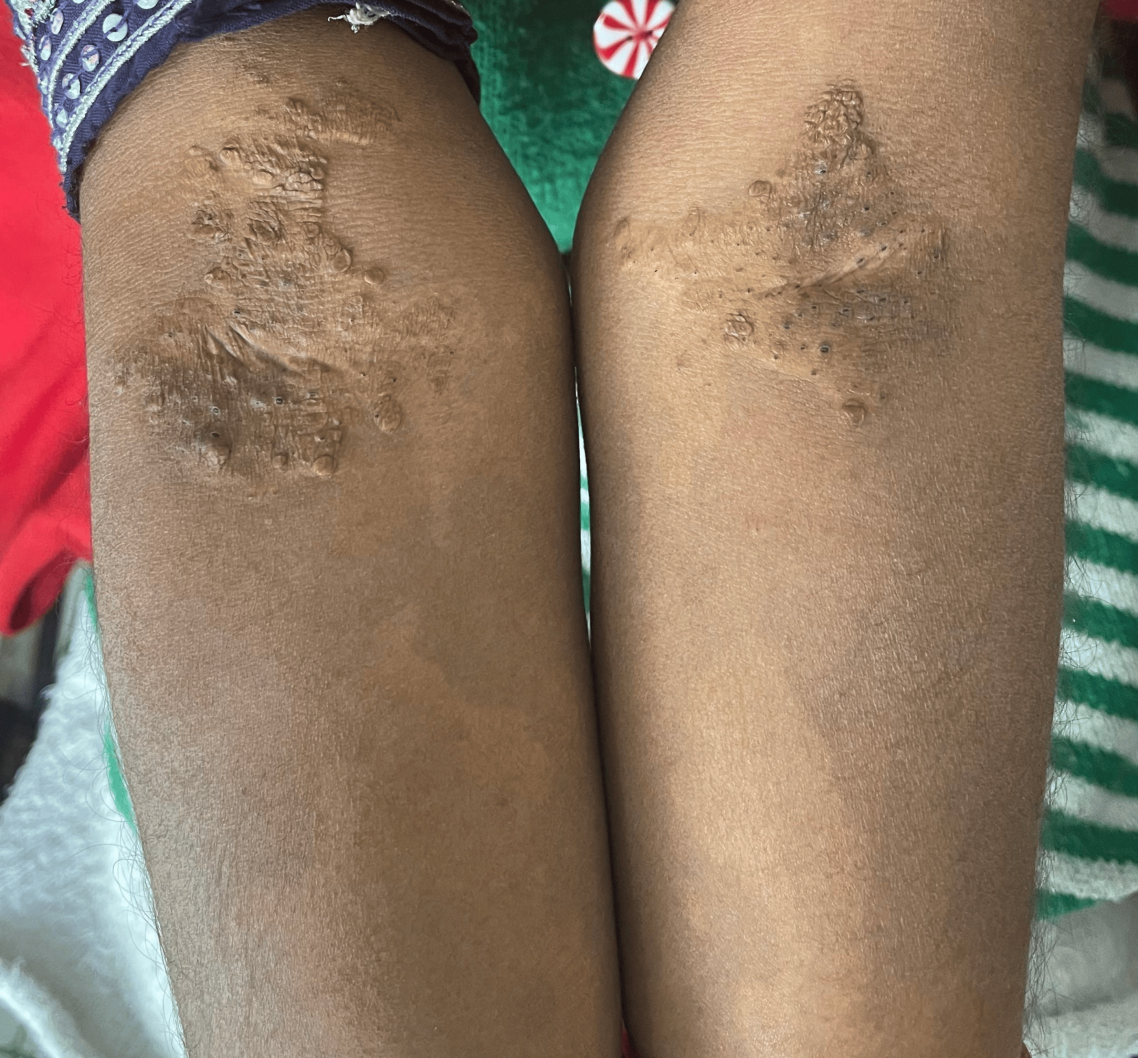
Well-demarcated yellowish plaques interspersed with multiple open and closed comedones, giving a mixed papular-comedonal appearance in the antecubital fossa bilaterally

On the anterior chest at the level of the clavicles bilaterally, there were large, irregular, yellow-colored plaques measuring approximately 5 x 7 cm (Figure 3). These lesions were painless and non-pruritic. A differential diagnosis of plane xanthomas, eruptive xanthomas, lipoid proteinosis, and pseudoxanthoma elasticum was considered. Given the clinical history and laboratory investigations, the lesions were identified as plane xanthomas.

**Figure 4:**
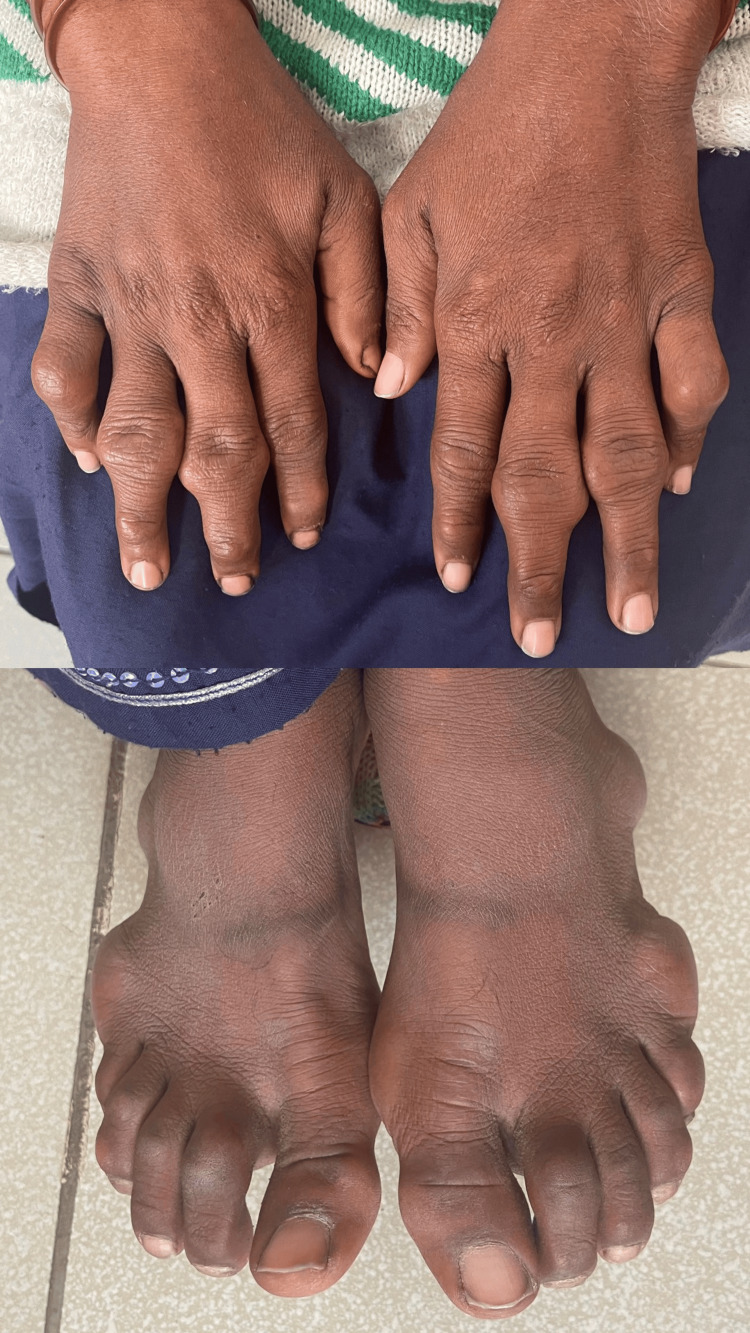
Firm, nodular swellings of varying sizes particularly over the interphalangeal joints, and the lateral borders of the feet mimicking rheumatoid nodules

The digits of both hands, particularly over the interphalangeal joints, and the lateral borders of the feet showed firm, nodular swellings of varying sizes (Figure 4). Some of these nodules were tender on palpation, and a flexural deformity was observed at the proximal interphalangeal joints of the little fingers bilaterally, limiting full extension. 

**Figure 5:**
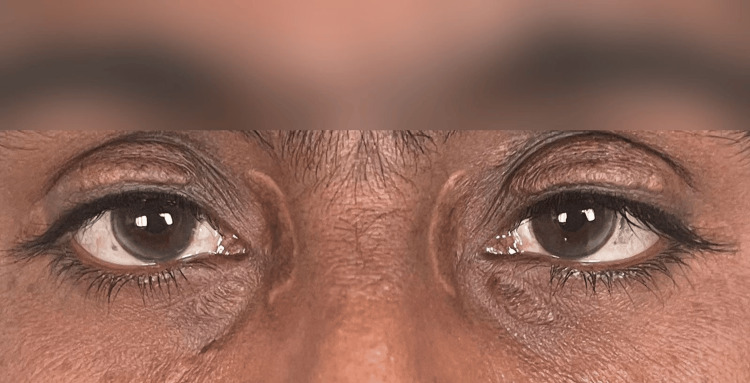
Bilateral corneal arcus

No signs of inflammation or secondary infection were noted. The rest of the skin examination, including scalp, nails, and mucosa, was unremarkable. Ocular examination revealed bilateral corneal arcus (Figure 5). Examination of the fundus did not reveal the presence of lipemia retinalis. The remainder of the systemic examination was within normal limits.

Routine laboratory investigations including complete blood count (CBC), urea, creatinine, electrolytes (UCEs), liver function tests, and creatine phosphokinase (CPK) were within normal limits. Screening for viral hepatitis showed negative results for HbsAg and anti-HCV antibodies. Glycated hemoglobin (HbA1c) was 5.2%, indicating no evidence of diabetes mellitus. Given the presence of joint deformities and nodular swellings, autoimmune markers including rheumatoid factor (RF), anti-nuclear antibody (ANA), and anti-cyclic citrullinated peptide antibody (anti-CCP) were also tested and found to be normal. Ultrasound examination in our patient did not reveal any evidence of synovitis, joint effusion, or power Doppler signal, effectively ruling out rheumatoid arthritis or other autoimmune inflammatory arthropathies. However, the lipid profile revealed severe dyslipidemia, with markedly elevated TC and LDL cholesterol levels, consistent with FH. The laboratory investigations are summarized in Table 1.

**Table 1:** Laboratory investigations of our patient HCV: Hepatitis C Virus; Anti-CCP antibodies: Anti-Cyclic Citrullinated Peptide Antibodies

Parameter	Value	Reference Range
Hemoglobin (Hb)	10.3 g/dL	12.0 – 15.5 g/dL
Total Leukocyte Count (TLC)	7.7 x10³/µL	4.0 – 11.0 x10³/µL
Platelet Count (PLTs)	294 x10³/µL	150 – 400 x10³/µL
Aspartate Aminotransferase (AST)	20 U/L	5 – 40 U/L
Alanine Aminotransferase (ALT)	25 U/L	7 – 56 U/L
Alkaline Phosphatase (ALP)	70 U/L	45 – 115 U/L
Total Bilirubin	0.8 mg/dL	0.1 – 1.2 mg/dL
Direct Bilirubin	0.2 mg/dL	0.0 – 0.3 mg/dL
Indirect Bilirubin	0.6 mg/dL	0.2 – 0.8 mg/dL
Urea	25 mg/dL	17 – 43 mg/dL
Serum Creatinine	0.6 mg/dL	0.5 – 1.1 mg/dL
Sodium	140 mmol/L	135 – 145 mmol/L
Potassium	4.2 mmol/L	3.5 – 5.1 mmol/L
Chloride	102 mmol/L	98 – 107 mmol/L
Bicarbonate	24 mmol/L	22 – 29 mmol/L
Hepatitis B Surface Antigen (HBsAg)	Non-reactive	Negative
Anti-HCV Antibodies	Non-reactive	Negative
Hemoglobin A1c (HbA1c)	5.20%	4.0 – 5.6%
Creatine Phosphokinase (CPK)	20 mcg/L	10 – 120 mcg/L
C-Reactive Protein (CRP)	2.3 mg/L	<5 mg/L
Erythrocyte Sedimentation Rate (ESR)	15 mm/hr	0 – 20 mm/hr
Total Cholesterol	654 mg/dL	<200 mg/dL
Triglycerides (TGL)	95 mg/dL	<150 mg/dL
High-Density Lipoprotein (HDL)	28.1 mg/dL	>50 mg/dL
Low-Density Lipoprotein (LDL)	607 mg/dL	<100 mg/dL
Rheumatoid Factor	Negative	<14 U/mL
Anti-CCP antibodies	Negative	<7 U/mL

The differential diagnosis of the joint deformities included chronic inflammatory arthritis, lipoid proteinosis, and other lipid storage disorders. However, the absence of inflammatory markers (CRP 2.3 mg/L, ESR 15 mm/hr) and negative autoimmune serology, coupled with severe isolated hypercholesterolemia, supports the diagnosis of FH with xanthomatous infiltration mimicking rheumatoid arthritis-like deformities. Radiological examination with PA views of the hands and feet demonstrated soft tissue swelling around interphalangeal and metacarpophalangeal joints, severe joint space narrowing at IP joints bilaterally, reduced bone density, and irregular articular surfaces particularly involving the bilateral little fingers. The wrist joints were normal. Foot X-rays revealed osteopenia, soft tissue swellings around IP, MTP, intertarsal, and ankle joints, acro-osteolysis of multiple toes, and reduction of joint space at IP joints. Echocardiography showed a normal ejection fraction of 60%, with no structural cardiac abnormalities.

Based on the Simon Broome criteria for FH, our patient fulfills the requirements for a definitive diagnosis of heterozygous FH [6]. She presents with markedly elevated TC (654 mg/dL) and LDL cholesterol (607 mg/dL), well above the diagnostic thresholds. Her mother has similar cutaneous lesions suggestive of hypercholesterolemia. Clinically, the patient exhibits characteristic tendon xanthomas manifested as nodular swellings on the digits and periocular xanthelasmas. A limitation in this case is the absence of genetic testing for FH. While the clinical and biochemical findings were suggestive of a lipid metabolism disorder, the lack of molecular confirmation limits the ability to definitively classify the type of hyperlipidemia. Genetic testing could have helped differentiate between monogenic forms of FH (such as LDLR, APOB, or PCSK9 mutations) and polygenic or secondary causes. This distinction has important implications for patient management, cascade screening of family members, and long-term cardiovascular risk assessment. However, the clinical and laboratory investigations satisfy the Simon Broome criteria, confirming the diagnosis (Table 2).

**Table 2:** Simon Broome criteria for our patient Source: [6] LDL-C: Low-Density Lipoprotein Cholesterol; FH: Familial Hypercholesterolemia; HoFH: Homozygous Familial Hypercholesterolemia

Parameter	Finding	Interpretation
Total Cholesterol	654 mg/dL	Very High (Supports Familial Hypercholesterolemia (FH)) Diagnosis)
Low-Density Lipoprotein Cholesterol (LDL-C)	607 mg/dL	Suggestive of Homozygous FH
Tendon Xanthomas	Present in the Patient	Meets Simon Broome "Definite FH" Criteria
DNA (Deoxyribonucleic Acid) Testing	Not Done	Not Essential for Simon Broome; Helps Confirm Type
Family History	Present in the Mother	Supports Possible FH
Likely Diagnosis	Homozygous FH (HoFH)	Based on Very High LDL-C + Tendon Xanthomas + FH Family History
Simon Broome Classification	Definite Familial Hypercholesterolemia	Clinical Criteria Met

The diagnosis of FH in this patient was further supported by the application of standardized diagnostic tools. Based on the Dutch Lipid Clinic Network (DLCN) criteria, the patient scored a total of 15 points; one point for a first-degree relative (mother) with similar cutaneous lesions, six points for the presence of tendon xanthomas, and eight points for an LDL cholesterol level exceeding 330 mg/dL (measured at 607 mg/dL) [7]. This score categorizes the patient as having definite FH. Similarly, applying the MEDPED (Make Early Diagnosis to Prevent Early Deaths) criteria, the patient aged 26 years with an LDL-C level of 607 mg/dL and a first-degree relative with probable FH, exceeds the LDL threshold of 190 mg/dL required for diagnosis in this age group, thereby confirming the diagnosis of FH by MEDPED standards as well (Table 3) [8].

**Table 3:** Diagnostic criteria application for familial hypercholesterolemia using DLCN and MEDPED criteria Source: [7,8] DLCN: Dutch Lipid Clinic Network; MEDPED: Make Early Diagnosis to Prevent Early Deaths; LDL-C: Low-Density Lipoprotein Cholesterol; FH: Familial Hypercholesterolemia

Parameter		Patient's Data	Points/Interpretation
DLCN			
LDL-C Level		607 mg/dL	+8 points
Tendon Xanthomas		Present (Digits, Periocular, Plaques)	+6 points
First-Degree Relative with Hypercholesterolemia		Mother with Similar Cutaneous Lesions	+1 point
Genetic Testing		Not Performed	0 points
Total Score		—	15 Points → Definite FH
MEDPED			
Age		26 Years	—
LDL-C Threshold for 1st-Degree Relative with FH		≥190 mg/dL	—
LDL-C Measured		607 mg/dL	Exceeds Threshold → FH Confirmed

Genetic testing to assess pathogenic variants in genes commonly implicated in FH, namely the LDLR, PCSK9, and apolipoprotein B100 (APOB), could not be performed in our patient due to limited local availability of testing facilities and financial constraints. Despite this limitation, cascade screening of first-degree relatives should be prioritized in future care, as early identification and treatment of affected individuals can significantly reduce cardiovascular morbidity and mortality associated with FH.

The patient’s management was multidisciplinary and included dietary counseling, lifestyle modification, and pharmacologic therapy with high-dose statins. The patient was referred to cardiology and neurology departments for comprehensive cardiovascular and cerebrovascular risk assessment, given her family history and severe hypercholesterolemia. Cardiovascular investigations such as ECG and echocardiography were planned to evaluate for ischemic changes and left ventricular function. In addition, non-invasive imaging, such as carotid Doppler ultrasound, was considered to assess the extent of vascular involvement. While the patient had no neurological complaints, carotid Doppler and, if indicated, brain MRI/MRA would be useful to screen for asymptomatic cerebrovascular disease given the increased risk of premature atherosclerosis in FH. Although PCSK9 inhibitors represent an effective advanced treatment option for FH, their limited availability and high cost in the local healthcare setting pose significant barriers to their use, making them a difficult option for therapy at present. The patient and her family were provided with genetic counseling, highlighting the autosomal dominant inheritance pattern of FH and the importance of cascade screening for at-risk relatives. Ongoing follow-up has been planned with regular lipid profiling and cardiac surveillance through echocardiography to monitor disease progression and therapeutic response.

## Discussion

This case holds substantial clinical relevance as, in addition to the classical manifestations of FH such as corneal arcus and tendon xanthomas, our patient exhibited tendon-related deformities that mimicked rheumatoid arthritis, a rare presentation of FH. These deformities are believed to result from chronic infiltration of lipid-laden (foamy) macrophages into the interphalangeal joints, leading to structural joint destruction and deformities over time. Unlike rheumatoid arthritis, however, these lesions are non-inflammatory in nature and are not associated with serological markers such as RF or anti-cyclic citrullinated peptide (anti-CCP) antibodies. Furthermore, systemic inflammatory markers typically remain within normal ranges. Therefore, in cases where patients present with nodule-like hand deformities, negative autoimmune serologies, and coexisting hyperlipidemia with a family history of premature cardiovascular disease, FH should be strongly considered in the differential diagnosis.

A similar clinical picture has been documented in the literature involving a young Bangladeshi girl who presented with extensive tendon xanthomas, corneal arcus, markedly elevated LDL-C and TC levels, and early-onset aortic valve disease [3]. She also developed rheumatoid-like deformities of the digits, attributed to progressive xanthomatous infiltration. However, apart from this case, no additional similar reports could be identified in the existing literature. To the best of our knowledge, this may represent the first report from Pakistan documenting such an atypical presentation of FH unless a systematic review confirms it. 

The extensive cutaneous involvement in our patient with xanthomas affecting the antecubital fossae, anterior chest, hands, feet, and periocular regions served as a crucial diagnostic clue. It is inferred that these xanthomas are composed of aggregates of lipids, including cholesterol, cholesterol esters, triglycerides, and phospholipids, surrounded by foamy macrophages [9]. Their development correlates with persistently elevated LDL-C levels in the bloodstream. Xanthomas typically arise during the second decade of life and present as firm, skin-colored to yellow papules or plaques [9]. Tendon xanthomas are highly characteristic of FH, with the Achilles tendon being the most frequently involved site, followed by the extensor tendons of the hands, feet, elbows, and knees [10]. Although they are generally slow to regress, timely initiation of lipid-lowering therapy can effectively halt their progression.

The presence of corneal arcus and xanthelasma palpebrarum in our patient further reinforced the clinical diagnosis of FH [11]. Corneal arcus is a notable clinical feature incorporated into the DLCN criteria, contributing a four-point score toward diagnostic classification [7]. Additionally, its occurrence, particularly in individuals under the age of 45, has been associated with an increased risk of premature cardiovascular disease, underscoring its prognostic as well as diagnostic significance in FH [3].

Despite the presence of characteristic cutaneous findings for several years, the delayed diagnosis in our patient highlights the existing lack of awareness and disparities in healthcare delivery in remote and rural areas of Pakistan. This case underscores the importance of recognizing physical signs such as tendon xanthomas, corneal arcus, and xanthelasma as valuable diagnostic clues for FH, particularly in settings where advanced diagnostic modalities like genetic testing are limited or unavailable [12]. The clinical identification of these physical stigmata remains a practical and indispensable component of the diagnostic process. Additionally, the use of validated diagnostic tools such as the Simon-Broome criteria, Dutch Lipid Clinic Network (DLCN) criteria, and the Make MEDPED criteria further strengthens the clinical suspicion and supports a provisional diagnosis in the absence of genetic confirmation, thereby facilitating timely intervention and reducing the risk of premature cardiovascular events [13].

This case highlights an important diagnostic challenge, as the patient presented with hand and foot deformities resembling those seen in rheumatoid arthritis, which were ultimately attributed to xanthomatous infiltration, a rare manifestation of FH. The coexistence of these atypical features alongside classical signs of FH underscores the need for a broad differential diagnosis when evaluating joint deformities, particularly in patients with underlying lipid disorders.

## Conclusions

This case highlights a rare and atypical manifestation of FH in a young woman who presented not only with classic cutaneous signs such as tendon xanthomas and corneal arcus but also with rheumatoid arthritis-like deformities of the hands and feet, an unusual finding in FH. The absence of autoantibodies and normal inflammatory markers distinguished these deformities from true inflammatory arthropathies like rheumatoid arthritis. This underscores the importance of considering FH in the differential diagnosis of non-inflammatory joint deformities, particularly in the presence of hyperlipidemia and a positive family history of premature cardiovascular disease. The case also emphasizes the diagnostic utility of clinical signs and scoring systems like the DLCN and MEDPED in low-resource settings where genetic testing is not feasible. This case underscores the importance of early diagnosis and comprehensive management of familial hypercholesterolemia to prevent serious cardiovascular complications, highlighting the clinical significance of recognizing atypical presentations for timely intervention. As the first reported case from Pakistan with such a presentation, it adds to the growing awareness of the diverse clinical spectrum of FH and the need for multidisciplinary care and long-term follow-up in affected individuals.
